# Comparison of transcriptome and metabolome analysis revealed cold-resistant metabolic pathways in cucumber roots under low-temperature stress in root zone

**DOI:** 10.3389/fpls.2024.1413716

**Published:** 2024-09-09

**Authors:** Shijun Sun, Yan Yang, Shuiyuan Hao, Ye Liu, Xin Zhang, Pudi Yang, Xudong Zhang, Yusong Luo

**Affiliations:** ^1^ Hetao College, Department of Agronomy, Bayannur, China; ^2^ Key Laboratory of Urban Agriculture, Ministry of Agriculture and Rural Affairs, Shanghai, China; ^3^ Hetao Green Agricultural Product Safety Production and Warning Control Laboratory, Hetao College, Bayannur, China; ^4^ Urat Middle Banner Green Industry Development Center, Bayannur, China; ^5^ Department of Horticulture, Hunan Agricultural University, Changsha, Hunan, China

**Keywords:** cucumber, root, cold-resistant, transcriptome, metabolome

## Abstract

**Introduction:**

Low ground temperature is a major factor limiting overwintering in cucumber cultivation facilities in northern alpine regions. Lower temperatures in the root zone directly affect the physiological function of the root system, which in turn affects the normal physiological activity of plants. However, the importance of the ground temperature in facilities has not attracted sufficient attention.

**Methods:**

Therefore, this study tested the cucumber variety Jinyou 35 under three root zone temperatures (room temperature, 20–22°C; suboptimal temperature, 13– 15°C; and low temperature, 8–10°C) to investigated possible cold resistance mechanisms in the root of cucumber seedlings through hormone, metabolomics, and transcriptomics analyses.

**Results and discussion:**

The results showed that cucumber roots were subjected to chilling stress at different temperatures. Hormone analysis indicated that auxin content was highest in the roots. Jasmonic acid and strigolactone participated in the low-temperature stress response. Auxin and jasmonate are key hormones that regulate the response of cucumber roots to low temperatures. Phenolic acid was the most abundant metabolite in cucumber roots under chilling stress. Additionally, triterpenes may play an important role in chilling resistance. Differentially expressed genes and metabolites were significantly enriched in benzoxazinoid biosynthesis in the room temperature vs. suboptimal temperature groups and the room temperature vs. low temperature groups. Most differentially expressed transcription factor genes in AP2/ERF were strongly induced in cucumber roots by both suboptimal and low-temperature stress conditions. These results provide guidance for the cultivation of cucumber in facilities.

## 1 Introduction

Cucumbers originated in the tropical rainforests of the southern foothills of the Himalayas and now exhibit a wide distribution, large cultivation area, high yield, and high economic value. According to FAO data (https://www.fao.org/zh), the area harvested in 2020 of cucumber reached 1,311,461 ha, and the yield reached 58,947.4 kg·ha^-1^ in China. Cucumber is a typical thermophilic plant with high-temperature requirements throughout its entire life cycle, especially for seedlings, which are more sensitive to low temperatures (Walters, 1989). In addition, different varieties of cucumber have different resistance to low temperature stress (Li et al., 2022). In northern China, cucumbers are often used for early spring and overwinter cultivation. The seedling stage or early planting date is often affected by low temperatures (such as the late spring coldness in March and April in Northern China), which affect plant growth and development, delay the fruit listing period, and may cause economic losses (Li et al., 2022).

At present, most horticultural facilities in northern China are relatively simple, and modern technology applications are limited. Therefore, crops grown in facilities are always grown under adverse environmental conditions, resulting in low yields, poor quality, and limited economic benefits (Sun et al., 2010). Environmental factors such as temperature, light, moisture, and CO_2_ concentration are of great significance for plant growth. Among these factors, root zone temperature plays a crucial role in plant growth (Sun et al., 2016). The adaptation range of cucumber root to temperature was narrow. Damage to root cells will affect the function of root and growth of aerial portion. Low-temperature root stresses can be categorized as chilling injury (cold injury above 0°C) and freezing injury (freezing injury below 0°C) (Jan et al., 2009; Chen et al., 2020). Previous studies have indicated that chilling injury affects the structure and function of cell membranes, enhancing membrane permeability and solute ion exudation, thereby changing the balance of ion concentrations inside and outside the cells (Bai et al., 2021). It also affects enzyme activity, leading to metabolic disorders (Zhao et al., 2021). While freezing injury can cause freezing inside and outside plant cells, salt toxicity, membrane protein degradation, and biofifilm system damage (Xu et al., 2023). In severe cases freezing injury may lead to the death of the whole plant (Saadati et al., 2021). Chilling and freezing injuries differ in their mechanisms and outcomes (Lijing Chen et al., 2014; Gong et al., 2020). In addition, chilling injuries account for the main issues occurring under low temperatures that must be solved in cucumber production facilities.

To develop a method for protecting cucumber crops from low-temperature stress and support future cultivation, we must first understand the mechanisms of low-temperature stress responses at the molecular level. In order to survive in low temperatures, plants transduce the cold signals into downstream components, thereby inducing appropriate defense mechanisms. Low temperature stress lead to the change of the lipids conformation and proteins in membrane, increases membrane permeability, inhibits ion transport and energy conversion pathways and cause damage to the ultrastructure of cells. Then the Ca^2+^ and other second messenger would be activates and amplified the signal in a cascade. These will eventually affecting the genes expression and biosynthesis of secondary metabolite involve in defense mechanisms modules (Zhu, 2016). The endogenous hormones plays an important role in response to low temperature stress in plant though participate in signal transduction and leads to expression of downstream genes (Xiang et al., 2017). Plant hormones, such as abscisic acid (ABA), brassinosteroid (BR), gibberellin (GA), and jasmonic acid (JA), have been reported to mediate plant adaptations to cold stress (Hu et al., 2013; Ding et al., 2015; Eremina et al., 2016; Li et al., 2017; Lantzouni et al., 2019).

With the rapid development of sequencing technology, transcriptomic and metabolomic analyses have been widely used to explore the molecular mechanisms by which plants cope with low-temperature stress (Bahrman et al., 2019; Xu et al., 2023). Transcriptomics and metabolomics analyses on 1-year-old branches of the cold-resistant cultivar Hanfu and the cold-sensitive apple cultivar Changfuji No. 2 revealed that Hanfu accumulated 4-aminobutyric acid, spermidine, and ascorbic acid to scavenge reactive oxygen species. The transcription factors apetala 2/ethylene responsive factor (AP2/ERF) and WRKY were strongly induced under freezing stress (Xu et al., 2023). Besides, the NAC family transcription factor were also involve in cold resistance. The CsNAC1 was induced by cold and ABA in both leaves and roots of citrus (*Citrus reshni*) (Mauch-Mani and Flors, 2009). A combination analysis on peach cultivars Donghe No.1 and 21st Century subjected to different temperatures (-5 to -30°C) for 12 h revealed that that soluble sugar, flavone, lignin biosynthesis-associated genes, and several key genes (e.g., COMT, CCR, CAD, PER, and F3’H) may play key roles in cold tolerance in peach (Li et al., 2023). Under low-temperature stress, the expression levels of some genes associated with plant hormones and MAPK pathways were significantly upregulated, and flavonoid metabolites were significantly enriched in waxy corn inbred lines N28 compared to those in N67. These genes and metabolites may help N28 improve cold resistance (Jiang et al., 2023). The During the cold treatment phase, the biological process changes mainly focus on antioxidant, and during the recovery period, a wide range of cold resistance reactions can be found, such as the accumulation of large amounts of amino acids. The combination analysis on 2 cold-resistant rice varieties (Nipponbare and 93–11) in different cold treatment stage reveled that during the metaphase of cold treatment, antioxidation-related compounds appeared earlier in Nipponbare, while ROS levels were higher in 93–11. Compared with CBF/DREB, ROS regulated genes were more active in Nipponbare stress response. Therefore, during the recovery period, metabolites related to cold resistance were more easily expressed in the cold-tolerant Nipponbare variety, while compounds related to aging were more easily accumulated in 93–11 (Zhang et al., 2016).

According to previous study, the best root zoom temperature for cucumber is 20–25°C. When the root zone temperature is lower than 15°C, the development and growth of cucumber root will be affected by low temperature (Xue et al., 2015). The aim of the present study was to evaluate the hormone change in the root of cucumber cultivar Jinyou 35 under three root zone temperatures (room temperature, 20–22°C; suboptimal temperature, 13–15°C; and low temperature, 8–10°C). The main metabolic pathways activated in response to chilling stress in cucumber roots were investigated using a combination of transcriptomics and metabolomics. Differentially expressed genes (DEGs) and their metabolites (DEMs) were identified. Identifying cold tolerance–related genes and analyzing the regulatory mechanisms of cold response in cucumbers using modern biological methods can provide theoretical support for the cultivation of cold-tolerant varieties.

## 2 Materials and methods

### 2.1 Plant materials

The ‘Jinyou 35’ cucumber cultivar was selected for analysis. ‘Jinyou 35’ is widely used in early spring cultivation for its good quality and cold resistance and it used to occupied more than 70% of the early spring cucumber cultivation area in China (around year 2010), and it is still widely used in Inner Mongolia province until now (https://www.taas.ac.cn) (Sun et al., 2016; Sun et al., 2018). The experiment was carried out in the Crop Cultivation Laboratory of Hetao College, Bayannaoer, Inner Mongolia Province (40°34′- 41°17′N, 107°6′-107°44′E). Three root zone temperatures were set: room temperature, 20–22°C; suboptimal temperature, 13–15°C; and low temperature, 8–10°C. A peat, vermiculite, and perlite mix with a volume ratio of 6:3:1 was used as the substrate for plant culture. Seedlings were planted in soil containing temperature controllers in mid-July 2022. The planting density was set at a line interval of 12 cm and a row interval of 18 cm.

To control the soil temperature, a temperature control line was laid at 15–18 cm depth below the soil, and the temperature-sensing probe was buried approximately 8 cm deep in the soil layer. Root zone temperatures were regulated from 22:00 to 6:00 the following day. After transplantation, cucumber seedlings were placed under low-temperature conditions. The room temperature setting (20–22°C) was used as the control (CK). The suboptimal (13–15°C) and low temperatures (8–10°C) were used as the treatment conditions. Each treatment group comprised 60 seedlings. Random sampling was used. Twenty days after transplantation, the root samples were immediately frozen in liquid nitrogen. The samples were sent to Wuhan Mai Tver Biotechnology Co., Ltd. to detect endogenous hormone content and conduct transcriptomic and metabolomic analysis. The root samples were named RR (root samples under room temperature conditions), SR (root samples under suboptimal temperature conditions), and LR (root samples under low-temperature conditions). Each test was performed in triplicate.

According to our previous studies, the root length, surf area, volume, tips and forks of cucumber under low temperature stress in the root zone is shown in **Table 1** (Sun et al., 2017).

**Table 1:** Effects of low root zone temperature on root morphology of cucumber (Sun et al., 2017).

Sample	Length	Surf Area	Root Volume	Tips	Forks
RR	315.71 ± 66.37aA	39.91 ± 4.38aA	0.59 ± 0.029aA	652 ± 97aA	3827 ± 593aA
SR	170.47 ± 67.71bB	25.86 ± 5.45bB	0.54 ± 0.032bB	305 ± 102bB	2150 ± 498bB
LR	82.34 ± 27.56cC	15.28 ± 4.69cC	0.27 ± 0.023cC	112 ± 38cC	836 ± 139cC

Lowercase letters indicates significant difference (0.05>P≥0.01) and uppercase letters represents extremely significant difference (P<0.01).

### 2.2 Determination of endogenous hormones

High performance liquid chromatography–grade acetonitrile and methanol were purchased from Merck (Darmstadt, Germany). Milli-Q water (Millipore, Bradford, USA) was used for all experiments. All standards were purchased from Olchemim Ltd. (Olomouc, Czech Republic) and IsoReag (Shanghai, China). Acetic and formic acids were purchased from Sigma-Aldrich (St. Louis, MO, USA). Standard stock solutions (1 mg/mL) were prepared in methanol. All stock solutions were stored at -20°C. The stock solutions were diluted with methanol to obtain working solutions prior to analysis.

After freezing in liquid nitrogen, root samples were ground into powder (30 Hz, 1 min) and stored at -80°C until needed. Root samples (50 mg) were weighed into a 2 mL plastic microtubes, frozen in liquid nitrogen, and dissolved in 1 mL methanol/water/formic acid (15:4:1, V/V/V). As internal standards for quantification, 10 µL of the internal standard mixed solution (100 ng/mL) was added to the extract. The mixture was vortexed for 10 min and centrifuged for 5 min (12000 r/min, and 4°C). Then, the supernatant was transferred to clean plastic microtubes, evaporated to dryness, dissolved in 100 μL 80% methanol (V/V), and filtered through a 0.22 μm membrane filter for further LC-MS/MS analysis (Cai et al., 2014; Floková et al., 2014; Niu et al., 2014; Li et al., 2016).

The root sample extracts were analyzed using an UPLC-ESI-MS/MS system (UPLC, ExionLC™ AD https://sciex.com.cn/; MS, Applied Biosystems 6500 Triple Quadrupole, https://sciex.com.cn/). The analytical conditions were as lay out in **Supplementary Table 1**.

Linear ion trap and triple quadrupole (QQQ) scans were acquired on a triple quadrupole–linear ion trap mass spectrometer (QTRAP), QTRAP^®^ 6500+ LC-MS/MS System equipped with an ESI Turbo Ion Spray interface, operating in both positive and negative ion modes and controlled by Analyst 1.6.3 software (Sciex). The ESI source operation parameters were as follows: ion source, ESI +/-; source temperature, 550°C; ion spray voltage, 5500 V (positive) and -4500 V (negative); curtain gas (CUR), 35 psi. Phytohormones were analyzed using scheduled multiple reaction monitoring (MRM). Data acquisition was performed using Analyst 1.6.3 software (Sciex). Multiquant 3.0.3 software (Sciex) was used to quantify all metabolites. Mass spectrometry parameters, including the declustering potentials (DPs) and collision energies (CEs) for individual MRM transitions, were performed with further DP and CE optimization. A specific set of MRM transitions were monitored for each period according to the metabolites eluted within this period (Pan et al., 2010; Cui et al., 2015; Šimura et al., 2018).

### 2.3 Metabolite profiling and data analyses

Root samples were freeze-dried using a vacuum freeze-dryer (Scientz-100F). The freeze-dried samples were crushed using a mixer mill (MM 400, Retsch) with zirconia bead for 1.5 min at 30 Hz. The lyophilized powder (50 mg) was powder in 1.2 mL of 70% methanol solution and vortexed for 30 s every 30 min for six rounds total. Following centrifugation at 12000 rpm for 3 min, the extracts were filtrated (SCAA-104, 0.22 μm pore size; ANPEL, Shanghai, China, http://www.anpel.com.cn/) before UPLC-MS/MS analysis.

The sample extracts were analyzed using an UPLC-ESI-MS/MS system (UPLC, ExionLC™ AD, https://sciex.com.cn/; MS, Applied Biosystems 4500 Q TRAP, https://sciex.com.cn/). The analytical conditions were as follows, UPLC: column, Agilent SB-C18 (1.8 µm, 2.1 mm * 100 mm); The mobile phase was consisted of solvent A (pure water with 0.1% formic acid) and solvent B (acetonitrile with 0.1% formic acid). Sample measurements were performed using a gradient program that employed starting conditions of 95% A and 5% B. Within 9 min, a linear gradient of 5% A and 95% B was programmed, and a composition of 5% A and 95% B was maintained for 1 min. Subsequently, the composition was adjusted to 95% A and 5.0% B within 1.1 min and kept for 2.9 min. The flow velocity was set as 0.35 mL per min; The column oven was set to 40°C; The injection volume was 4 μL. The effluent was alternately connected to an ESI-triple quadrupole–linear ion trap-MS (QTRAP)-MS.

The ESI source operation parameters were as follows: source temperature, 550°C; ion spray voltage, 5500 V (positive) and -4500 V (negative); ion source gas I (GSI), gas II (GSII), and CUR at 50, 60, and 25 psi, respectively; and high collision-activated dissociation. QQQ scans were acquired in the MRM experiments with a collision gas (nitrogen) in the medium. The DP and CE for individual MRM transitions were determined with further DP and CE optimization. A specific set of MRM transitions was monitored for each period according to the metabolites eluted within this period.

Unsupervised principal component analysis (PCA) was performed using the prcomp statistical function in R (www.R-project.Org/). The data were unit variance scaled before unsupervised PCA.

Hierarchical cluster analysis (HCA) results of samples and metabolites were presented as heatmaps with dendrograms, whereas Pearson correlation coefficients (PCC) between samples were calculated using the cor function in R and presented only as heatmaps. Both HCA and PCC were performed using the R package, Complex Heatmap. For the HCA, the normalized signal intensities of the metabolites (unit variance scaling) were visualized as a color spectrum.

For two-group analysis, differential metabolites were determined by VIP (VIP ≥ 1) and absolute Log2FC (|Log2FC|≥ 1.0). VIP values were extracted from the OPLS-DA results, which also contained score plots and permutation plots, and were generated using the R package MetaboAnalystR. The data were log-transformed (log2) and mean-centered before OPLS-DA. A permutation test (200 permutations) was performed to avoid overfitting.

Identified metabolites were annotated using the KEGG Compound Database (http://www.kegg.jp/XXXeg/compound/), and annotated metabolites were mapped to the KEGG Pathway Database (http://www.kegg.jp/XXXeg/pathway.html). Pathways with mapped significantly regulated metabolites were then fed into metabolite set enrichment analysis, and their significance was determined using hypergeometric test p-values.

### 2.4 RNA-seq and data analysis

Root samples were immediately frozen in liquid nitrogen and stored at −80°C. Total RNA was extracted from roots using the RNAprep Pure Polyphenol Plant Total RNA Extraction Kit (TIANGEN, China). A cDNA library was constructed and sequenced on an Illumina HiSeq4000 system supplied by Mateware. The adapters and low-quality sequences were removed using Fastp with default parameters, and clean reads were mapped to the reference genome of the ChineseLonggenomev3.fa.gz using HISAT2. The number of mapped reads and transcript lengths were normalized. The fragments per kilobase million (FPKM) were used as indicators of transcript or gene expression. The Pearson correlation coefficient and PCA were used to evaluate the correlation and repetitiveness among samples. Differential expression analysis was performed using DESeq2 (|log2- old change| ≥1, and FDR < 0.05) to obtain differentially expressed genes between two samples. Heat maps and Venn diagrams were drawn using the Tbtools software. Three biological replicates were used for each root sample.

Nonredundant transcript sequences identified as genes were further analyzed by Gene Ontology (GO) annotation (http://www.ge-neontology.org/) to identify GO terms among DEGs with significant differences, and the Kyoto Encyclopedia of Genes and Genomes (KEGG) database (http://www.genome.jp/XXXeg/) was used to identify significantly enriched pathways.

To verify the accuracy of RNA-seq data, q-RT-PCR validation was performed using frozen root samples. First, strand c DNA was synthesized using TAKARA PrimeScript™ RTMaster Mix (Perfect Real Time). Real-time quantitative PCR was performed using a QTOWER Real-time fluorescence quantitative PCR instrument (ANALYTIKJENA, Germany) using Talent qPCR PreMix (SYBR Green). The cucumber actin gene was used as an internal control. Each reaction was performed in triplicate with are action volume of 20 μL. Cycling parameters were 96°C for 10 min, 40 cycles of 95°C for 15 s, 58°C for 20 s and 72°C for 30 s, final extension at 72°C for 10 min. Quantitative data were calculated as 2^−△△^CT. Primer sequences are listed in **Supplementary Table 2**. Each test was performed in triplicate.

### 2.5 Statistical analyses

Each treatment was performed in triplicate, and the data are presented as the mean ± standard deviation. Significant differences (P < 0.05) were evaluated using SPSS Statistics (version 26.0; SPSS Inc., Chicago, IL, USA) and one-way analysis of variance with Duncan’s test. The comparison groups of endogenous hormones, metabolites, and RNA-seq data were divided into RR vs. SR, RR vs. LR, and SR vs. LR groups.

## 3 Results

### 3.1 Endogenous hormone responses of cucumber roots to chilling stress

**Figure 1:**
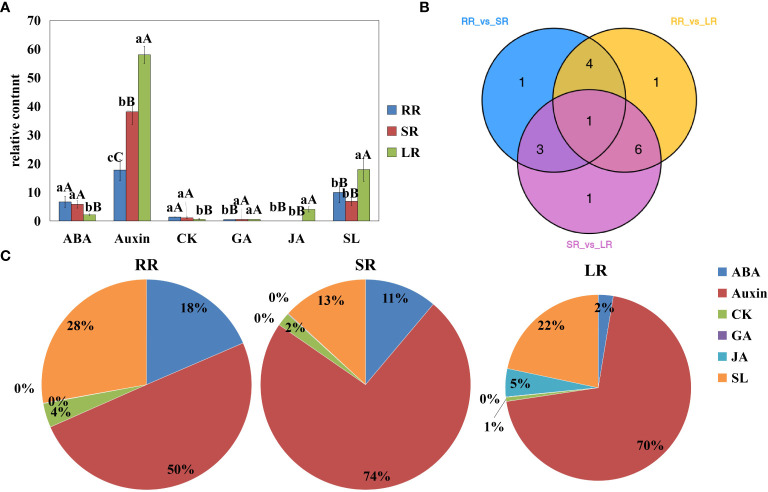
Content of differentially accumulated endogenous hormones **(A)**, Venn diagram **(B)**, and pie chart **(C)** RR, SR and LR. Values are average on three replicates. Significant differences are indicated by letters, lowercase letter indicates significant difference (0.05>P≥0.01), uppercase letter represents extremely significant difference (0.01>P≥0.001). RR: The cucumber root samples under room temperature condition; SR: The cucumber root samples under suboptimal temperature condition LR: The cucumber leaf samples under low temperature condition. The same below.

In this study, roots of the cucumber cultivar Jinyou 35 were subjected to different degrees of low temperature stress. We identified 17 differentially accumulated endogenous hormones among RR vs. SR, RR vs. LR, and SR vs. LR (**Figure 1**). Detailed information on differentially accumulated endogenous hormones is listed in **Supplementary Table 3**. Differentially accumulated endogenous hormones can be classified into six categories. As the temperature decreased, ABA and cytokinin (CK) content decreased, while auxin, GA, and JA content increased. Strigolactone (SL) content increased in SR and decreased in LR compared with RR. The hormone with the highest accumulation in cucumber roots was auxin. The proportions of SR (74%) and LR (74%) were higher than that of RR (50%), indicating that auxin is important not only in root development, but also in response to chilling injury. The accumulation trend of CK was opposite to that of auxin, indicating that the relationship between auxin and CK is stress resistant under low-temperature stress. Auxins can increase the GA levels in plants. The accumulation trend of GA was similar to that of auxin but was much lower in content. The ABA content decreased as temperature decreased, and the ratio decreased from 18% in RR to 2% in LR. There was a stress-resistance relationship between GA and ABA in cucumber roots under low-temperature stress. The JA content increased sharply in LR and was hardly detected in RR and SR, indicating that JA might play an important role in low-temperature stress. The SL content in LR was significantly higher than in RR and SR; however, the ratio in LR (22%) was lower than that in RR.

Venn diagrams show that 9, 12, and 11 differentially accumulated endogenous hormones were obtained for RR vs. SR, RR vs. LR, and SR vs. LR, respectively. These hormones may play key roles in chilling stress response. One hormone (2MeSiP) was accumulated in all groups (**Figure 1B**). These hormones may be associated with low root zone temperature responses in cucumber roots.

### 3.2 Metabolite identification in cucumber root

To further study the difference in the response of cucumber to suboptimal and low temperatures compared to that under room temperature, metabolomic analyses were performed. LC-MS/MS was used for targeted metabolomic analysis. MRM detection, PCA, and correlation analyses showed that the data quality met the requirements for subsequent analyses (**Supplementary Figures S1A-D**). Of the 995 metabolites screened for each comparison group under the VIP, FC, and P-value triple screening conditions, 119 were DEMs in RR vs. SR (75 upregulated and 44 downregulated), 152 were DEMs in RR vs. LR (96 upregulated and 56 downregulated), and 120 were DEMs in SR vs. LR (60 upregulated and 60 downregulated) (**Supplementary Figures S1F-H**). To better understand the metabolic response mechanisms of cucumber roots under low-temperature stress, we analyzed the DEMs identified in each comparison group. The Venn diagram analysis further showed there were 55 DEMs common to RR vs. SR and RR vs. LR. Most DEMs are flavonoids and phenolic acids that improve the suboptimal and low-temperature tolerance of plants by increasing the activity of antioxidant enzymes. Five DEMs were common to all three groups. All five DEMs were flavonoids, with two isoflavones and three flavones identified.

**Figure 2:**
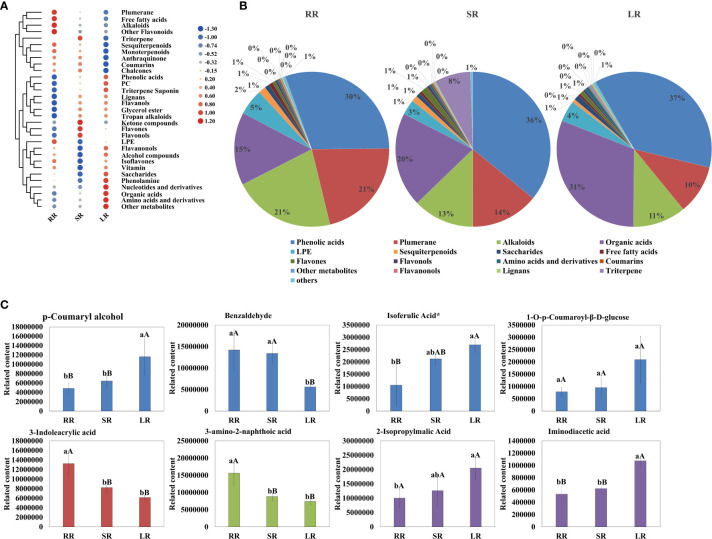
Cluster heatmap **(A)** and pie chart **(B)** of metabolites classified by Class II. Main content of phenolic acids, plumerane, alkaloids, and organic acids **(C)**.

As shown in **Figure 2**, the contents of phenolic acids, plumerane, alkaloids, and organic acids were significantly higher than those of the other metabolites. The detail information of metabolites in phenolic acids, plumerane, alkaloids, organic acids and triterpene class was shown in **Supplementary Table 4**. Phenolic acid was the most abundant compound. The percentages of SR (36%) and LR (37%) were almost the same and higher than that of RR (30%). The main phenolic acids that accumulated in the cucumber roots were p-Coumaryl alcohol, benzaldehyde, and isoferulic acid* (**Figure 2C**), the relative contents of which are listed in **Supplementary Table 4**. The downward trends of plumerane (21% in RR, 14% in SR, and 10% in LR) and alkaloids (21% in RR, 13% in SR, and 11% in LR) with temperature were almost the same. The main components of plumerane were 3-Indoleacrylic acid, indole-3-carboxaldehyde, and methoxyindoleacetic acid. The alkaloids used were 3-amino-2-naphthoic acid and cyclohexylamine. Organic acids (15% in RR, 20% in SR, and 31% in LR) increased sharply with decreasing temperature, especially in SR and LR. 2-Isopropylmalic acid, iminodiacetic acid, and JAs are the main organic acids accumulated in cucumber roots. Additionally, the triterpene (isomangiferolic acid and ursolic acid) content in SR was significantly higher than in RR (269 times) and LR (195102 times), which may play an important role in the chilling resistance process.

### 3.3 Transcriptomic analysis in cucumber root

To further study the difference in response to suboptimal and low temperatures compared to that under room temperature in cucumber, transcriptomic analyses were performed. We prepared nine cDNA libraries. The Q20 and Q30 values of each cDNA library were greater than 97% and 92%, respectively; the base error rate was 0.03%, the average G/C content was 42.61%, and the average base number was 6.56 Gb (**Supplementary Table 5**). The proportion of clean reads that mapped to the reference genome was > 95%. In addition, the correlation coefficients between the samples were calculated based on the FPKM values of all genes in each sample, and PCA was performed. The results showed that the square of the correlation coefficient (R2) was greater than 0.92 and that the samples were scattered between groups and gathered within them (**Supplementary Figures S2A, C**). Cluster analysis revealed a clear separation between the samples, whereas replicate samples from each treatment clustered together (**Supplementary Figure S2B**). Three biological replicates were analyzed for each cucumber root sample group. Most DEGs were found in the RR vs. LR comparison (1136; 761 upregulated and 375 downregulated genes), followed by those in SR vs. LR (366; 287 upregulated and 79 downregulated genes); RR vs. SR had the lowest number of DEGs (189; 150 upregulated and 39 downregulated genes). Interestingly, the number of upregulated DEGs in each comparison group was higher than that of the downregulated DEGs (**Supplementary Figure S2D**). In addition, Venn diagram analysis revealed 141 common DEGs between the RR vs. SR and RR vs. LR groups (**Supplementary Figure S2E**). In total, 256 common DEGs were identified between RR vs. SR and SR vs. LR. Only six DEGs were common between the RR vs. SR and SR vs. LR groups. Ten common DEGs were expressed in all three groups.

### 3.4 Comparative analysis of DEGs and DEMs in cucumber root under low-temperature stress

**Figure 3:**
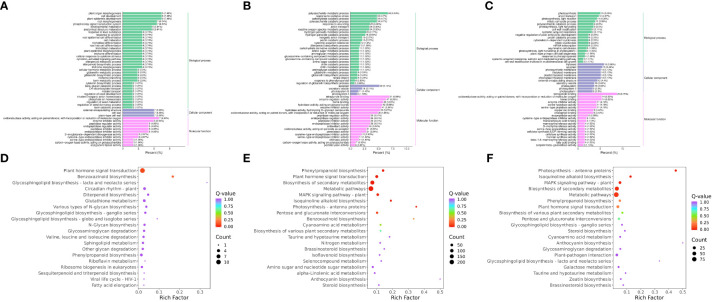
GO enrichment of DEGs in RR vs. SR **(A)**, RR vs. LR **(B)** and SR vs. LR **(C)**. KEGG enrichment of DEGs in RR vs. SR **(D)**, RR vs. LR **(E)** and SR vs. LR **(F)**.

To compare the low-temperature response differences under different temperature conditions, the enrichment of DEGs and DEMs was analyzed in the three comparison groups. The GO enrichment results showed that the DEGs in RR vs. SR were significantly enriched in plant organ morphogenesis (GO:1905392), cell development (GO:0048468), and plant epidermis development (GO:0090558) (**Figure 3A**). The DEGs in RR vs. LR were significantly enriched in tetrapyrrole binding (GO:0046906), polysaccharide metabolic processes (GO:0005976), and enzyme regulator activity (GO:0030234) (**Figure 3B**). The DEGs in SR vs. LR were significantly enriched in tetrapyrrole binding (GO:0046906), thylakoids (GO:0009579), and apoplasts (GO:0048046) (**Figure 3C**).

KEGG enrichment analysis of the DEGs showed that 118 DEGs were enriched in 47 pathways in RR vs. SR, while 1 043 DEGs were enriched in 111 pathways in RR vs. LR (**Figures 3D, E**). Plant hormone signal transduction (ko04075) and benzoxazinoid biosynthesis (ko00402) were significantly enriched in the two comparison groups, indicating that cucumber roots responded to suboptimal and low-temperature stress through these two basic metabolic pathways. In total, 392 DEGs were enriched in 82 pathways in SR vs. LR (**Figure 3F**). Photosynthesis-antenna proteins (ko00196), isoquinoline alkaloid biosynthesis (ko00950), the MAPK signaling pathway (ko04016), phenylpropanoid biosynthesis (ko00940), pentose and glucuronate interconversions (ko00040), and cyanoamino acid metabolism (ko00460) were significantly enriched in the RR vs. LR and SR vs. LR comparison groups. The results showed that cucumber roots might respond exclusively to low-temperature stress through these metabolic pathways.

**Figure 4:**
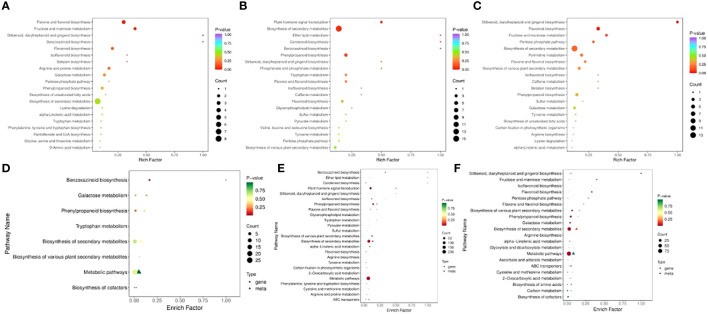
KEGG enrichment of DAMs in RR vs. SR **(A)**, RR vs. LR **(B)**, and SR vs. LR **(C)**. The KEGG enrichment of DEGs and DAMs in RR vs. SR **(D)**, RR vs. LR **(E)**, and SR vs. LR **(F)**.

In RR vs. SR, 52 DEMs were enriched in 27 pathways, while 75 DEMs were enriched in 33 pathways in RR vs. LR (**Figures 4A, B**). Benzoxazinoid biosynthesis (ko00402); stilbenoid, diarylheptanoid, and gingerol biosynthesis (ko00945); flavone and flavonol biosynthesis (ko00944); flavonoid biosynthesis (ko00941); and isoflavonoid biosynthesis (ko00943) were significantly enriched in the two comparison groups, indicating that these DAMs may be key metabolites that respond to suboptimal and low-temperature stress in cucumber roots. For RR vs. SR, the DEGs and DEMs were significantly enriched in the benzoxazinoid biosynthesis pathway. In SR vs. LR, 80 DEMs were enriched in 33 pathways (**Figure 4C**). Stilbenoid, diarylheptanoid, gingerol, and flavonoid biosynthesis were the main pathways enriched in this group.

Association analysis of transcriptomics and metabolomics allows the prediction of changes in metabolites at the transcriptional level and verifies the results of gene transcription at the metabolic level. Also it further analyzing the relationship between metabolic and transcriptional spectra and the metabolic mechanisms of various biological systems of plant (Cavill et al., 2016). The DEGs and DEMs mapped to the KEGG pathway database were compared to obtain information on common pathways. In the RR vs. SR, RR vs. LR, and SR vs. LR comparison groups, 8, 28, and 21 pathways were co-enriched by DEGs and DEMs, respectively. For RR vs. SR, DEGs and DEMs were significantly enriched in the benzoxazinoid biosynthesis pathway (**Figure 4D**). In RR vs. LR, DEGs and DEMs were significantly enriched in benzoxazinoid biosynthesis, phenylpropanoid biosynthesis, plant hormone signal transduction, and other pathways (**Figure 4E**). In SR vs. LR, DEGs and DEMs were significantly enriched in some biosynthesis pathways such as stilbenoid, diarylheptanoid, gingerol, flavonoid, and phenylpropanoid biosynthesis (**Figure 4F**). In summary, these common enrichment pathways may be the transcriptomic and metabolic bases for low-temperature resistance in cucumber roots.

### 3.5 DEGs and DAMs involved in phenylpropanoid biosynthesis

**Figure 5:**
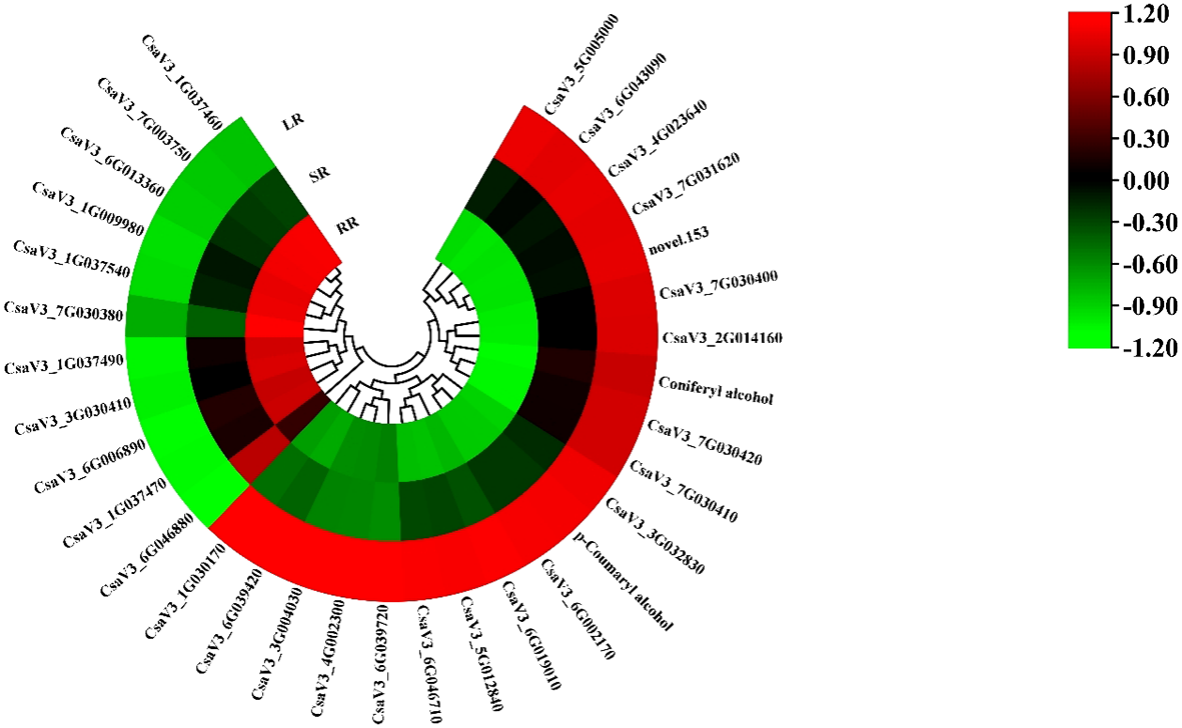
The heatmap of DEGs and DAMs involved in phenylpropanoid biosynthesis.

The phenylpropanoid biosynthesis pathway exists widely in higher plants and leads to the production of many secondary metabolites related to low-temperature stress, such as flavonoids, terpenoids, and phenolic acids. Most DEGs in the phenylpropanoid biosynthesis pathway increased with decreasing temperature (**Figure 5**). The other ten genes showed the opposite trend. The cytochrome P450 gene, CsaF5H, showed the highest expression in the SR. Two phenolic acids, DAMs, p-Coumaryl alcohol and coniferyl alcohol, are involved in phenylpropanoid biosynthesis. CsaPER52 (CsaV36G002170), which regulates the conversion of p-Coumaryl alcohol to p-Hydroxyphenyl lignin, is highly related to the content of p-Coumaryl. While CsaPER5, which participates in the conversion of coniferyl alcohol to guaiacyl lignin, is highly related to coniferyl alcohol content. The increasing trend of these DAMs and DEGs may promote cucumber root response to low-temperature stress.

### 3.6 Key transcription factors associated with low-temperature stress

**Figure 6:**
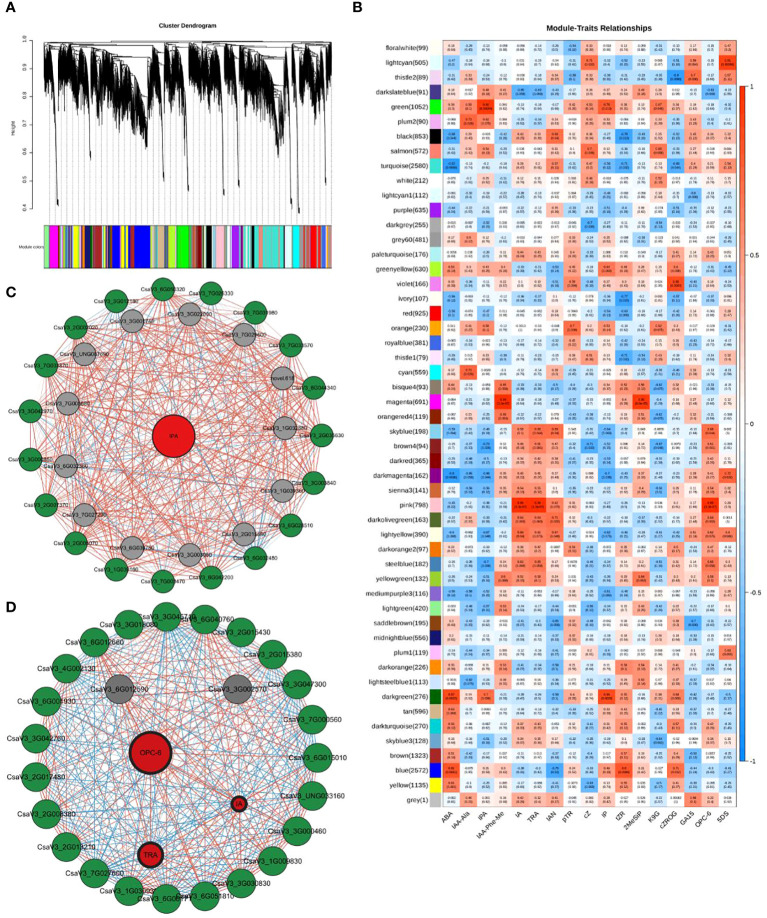
WGCNA results. **(A)** Tree maps of 22,551 genes were constructed by hierarchical clusters with topological overlap dissimilarity. Each gene was represented by leaves in the tree branch. The coexpression distance between two genes represents the height on the y-axis. The first and second lines below the tree indicate the module members identified by the dynamic tree using the cutting method and the combined dynamic tree identity with a 0.85 merge threshold, respectively. The main branches comprise 52 coexpression modules. **(B)** Relationships between differentially accumulated endogenous hormones in cucumber roots (column) and module characteristic genes (MEs, row). The color indicates intensity and direction. The numbers in parentheses are partial Pearson correlations and corresponding P-values. **(C)** Subnetwork of IPA and putative transcription factors (TFs) and structural genes related to Plant hormone signal transduction from the green module. **(D)**: Subnetwork of three plant hormones and structural genes related to Plant hormone signal transduction and putative TFs and from the pink module. Red circles denote metabolite, green circles denote pathway genes, and gray circles denote TFs. The lines between the two dots indicate their interactions. The colors of lines represent the positive or negative relationship between two genes or metabolite and gene. The width of lines represents the strength of correlations between the two genes; the wider the stronger and the thinner the weaker the interactions. The width of label lines represents the FC between two inbred lines; the wider the larger stronger and the thinner the smaller the multipliers.

Weighted gene co-expression network analysis (WGCNA) was used to identify the characteristic genes of a module, key genes in the module, association between modules, and sample phenotypes (Langfelder and Horvath, 2008). Using WGCNA, all genes in the nine cucumber root samples (RR, SR, and LR with three biological replicates) were divided into 52 coexpression modules (**Figure 6A**, **Supplementary Table S6**). We identified 1052 coexpressed genes in the green module, which had a significantly positive correlation with IPA (r = 0.92, P = 0.00044), while 798 coexpressed genes were identified in the pink module and had a positive correlation with IA (r = 0.99, P = 3.3e‐07), TRA (r = 0.99, P = 3.3e‐07), and OPC-6 (r = 0.99, P = 3.3e‐07) (**Figure 6B**). The gene expression patterns of the green module were significantly correlated with the hormone with the highest content in the SR group, which might relate to suboptimal temperature stress. The gene expression patterns of the pink module were significantly correlated with hormone with the highest content in the LR group, which might be related to low-temperature stress.

When PCC was set as ≥0.90 or ≤−0.90, 1052 and 798 DEGs were used to construct a coexpression network of green and pink modules, respectively (**Supplementary Table S7**). The expression levels of 37 genes from the green and pink modules that were highly correlated with hormone content in cucumber roots may be regarded as highly connected genes (hub genes) and were involved in two categories: plant hormone signal transduction (16) and transcription factors (TFs) (21) (**Figures 6C, D**; **Supplementary Table S8**).

According to the expression patterns of DEGs in the plant hormone signal transduction pathway, some TFs may be involved in the regulation of hormone content in cucumber roots, resulting in responses to low-temperature stress. Previous studies have demonstrated that AP2/ERF, bHLH, WRKY, and other genes play important roles in the response of plants to low-temperature stress (Yonghong Li et al., 2023; Xu et al., 2023). In green and pink modules, 21 differentially expressed TF genes (DETFs) were identified in the gene coexpression network.

The DETF in the green module were involved in three categories, the AP2/ERF (CsaV32G035630, CsaV33G003840, CsaV36G028510, CsaV36G032480, CsaV36G042200, CsaV37G003470), bHLH (CsaV31G039160, CsaV32G005070, CsaV32G007370, CsaV33G000850, CsaV33G042970, CsaV37G003870) and WRKY family (CsaV32G002020, CsaV33G012180, CsaV36G050320, CsaV37G026330, CsaV37G031980, CsaV37G033570, and CsaV36G044340). The DETF in the pink module belong to the AP2/ERF family (CsaV33G002570 and CsaV36G012590). These results indicated that the WRKY family and CBF1 genes mainly responded to suboptimal temperature stress. Different DETFs in the AP2/ERF family may regulate the response to suboptimal and low-temperature stress in cucumber roots.

In addition, in the plant hormone signal transduction pathway, 18 DEGs (one AUX1, four AUX/IAA, three ARF, 3 GH3 and seven SAUR) participated in auxin signal transduction (**Figure 6D**). Seven DEGs (2 B-ARRs and 5 A-ARRs) affected CK signal transduction. Seven DEGs (two GID1, three DELLA, and two PIF3) regulated the GA signal transduction. Eight DEGs (four BRI1, one BKI1, one BSK, one TCH4, and one CYCD3) were associated with BR signal transduction. One and two DEGs (TGA and two JAZ) were associated with salicylic acid and jasmonic acid (JA) signal transduction, respectively. Interestingly, no DEGs were detected during ABA or ethylene signal transduction. This indicates that these two hormones may have little effect on the response to chilling stress in cucumber roots.

**Figure 7:**
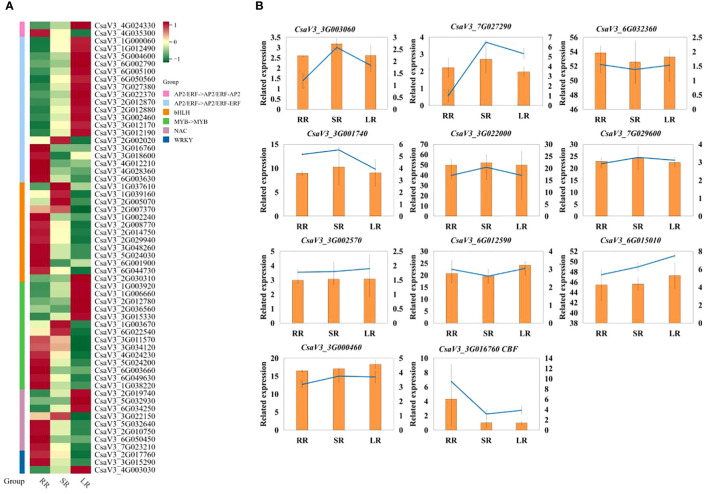
**(A)** Heat map of DETFs in AP2, bHLH, MYB, NAC and WRKY family. **(B)** q-RT-PCR result.

To further analyze the TFs related to the response to low-temperature stress in cucumber roots, we created a heat map of DETFs in the AP2, bHLH, MYB, NAC, and WRKY families (**Figure 7A**). Most DETFs were upregulated with a decrease temperature in AP2 family. Additionally, DREB/CBF (C-repeat binding/dehydration-responsive element-binding protein, *CsaV33G016760*) in the AP2/ERF-ERF Subfamily was downregulated in SR and LR compared to RR. The other DREB (dehydration-responsive element-binding protein, CsaV32G002020) gene was upregulated in SR and downregulated in LR, whereas in the bHLH and WRKY families, most DETFs were downregulated in LR compared with RR. The upregulated DETF in WRKY family is WRKY75 (*CsaV34G003030*). Half of the DEGs were downregulated in the MYB and NAC families. The qRT-PCR results for the key genes are presented in **Figure 7B**. The results of q-RT-PCR were basically consistent with those of transcriptome sequencing, which proved that the sequencing was reliable.

## 4 Discussion

Under suboptimal low temperature stress, the biomass of cucumber root decreased significantly compared with room temperature. Increasing evidence suggests that specific hormones play an important role in plant responses to low-temperature stress, including ABA, JA, salicylic acid (SA), GA, brassinolactone (Br), CK, ethylene and so on (D. Chen, Wang, Yue, & Zhao, 2016). Auxin is a common factor in most hormone regulatory networks (Chen et al., 2016; Xiao, et al., 2018). It is not only involved in the whole process of plant development but also participates in regulating plant adaptability to environmental change (Chen et al., 2016; Zhang et al., 2020). In cucumber root, auxin plays a key role in maintaining the root growth (Su and Masson, 2019). In our study, auxin accumulated abundantly under low-temperature conditions, and its content was higher than that of all other hormones. Studies on ABA have demonstrated that it is an important regulator of the whole plant life cycle and is involved in regulating many abiotic stress responses (Li et al., 2022). However, in our study, ABA was not the main hormone that determined the response to chilling stress in cucumber roots. JA also regulates the response of cucumber roots to low-temperature stress. These two hormones are key regulators of root development. Studies on the relationship between JA and low-temperature stress have mainly focused on fruits. The mechanism by which JA signal transduction responds to chilling stress in cucumber roots requires further investigation. Studies on cucumber cold-resistant cultivars Zhongnong37 and cold-sensitive cultivars Shuyanbailv under low-temperature condition reveal that the IAA, ABA, and JA content in cold-resistant cultivars was higher than cold-sensitive cultivars under three low-temperature treatment conditions (15–10, 12–8, and 9–5°C) (Amin et al., 2022). After H2S treatment, a sharp increase in endogenous IAA content was observed in cucumbers, which enhanced tolerance to cold stress. Conversely, NPA application significantly compromised cucumber defense against cold by decreasing endogenous IAA and H2S contents (2020). These studies indicate that auxin plays a key role in cold stress and enhances the resistance to cold injury in cucumbers.

The contents of phenolic acids, plumerane, alkaloids, and organic acids were significantly higher than those of other metabolites accumulated in the cucumber roots. Phenolic acid was the most abundant compound. The accumulation of soluble phenolic acids is also involved in cold stress responses in plants. In rice, the phenolic constituents of phenols, polyphenols, flavonoids, and phenolic acids have different bound forms in Japonica and Indica subtypes, and these may have a positive role under chilling stress (Rayee et al., 2020). Plumerane and alkaloids were positively correlated with temperature, whereas organic acids were negatively correlated with temperature. Alkaloids are secondary metabolites produced by the interaction between plants and the external ecological environment. As chemical defense substances, alkaloids play an important role in resisting the influence of adverse environment such as high-temperature and drought (Li et al., 2012). But there are few study on function of alkaloids in low temperature resistance in cucumber root. Organic acids affect plant resistance during exposure to low temperature. The accumulation of fumaric acid and azelaic acid were crucial for response to cold stress in *Arabidopsis* (Xu et al., 2011; Dyson et al., 2016). In our study, 2-Isopropylmalic acid, iminodiacetic acid, and JA were the main organic acids accumulated in cucumber roots, indicating that they were important for the response of cucumber roots to cold stress. Additionally, triterpenes may play an important role in cold resistance. Benzoxazinoid and phenylpropanoid biosyntheses were the main differential pathways in response to chilling injury in cucumber roots and are both involved in plant abiotic stress responses. The final product of phenylpropanoid biosynthesis is coumaric acid, which is the precursor of downstream pathways, such as flavonoid-related pathways and lignin synthesis. This has also been introduced by temperature (Dong and Lin, 2020). Benzoxazinoid biosynthesis has garnered significant research attention for its role in defensive process (disease resistance, insect resistance,etc) and allelopathy (Gao et al., 2017). However, its relationship with the low-temperature stress requires further investigation.

TFs play crucial roles in plant growth, development, and adaptation to stress. AP2/ERF and WRKY play important roles in plant responses to low-temperature stress (Xu et al., 2023). Therefore, this study mainly analyzed the expression patterns of AP2/ERF and WRKY and found that, WRKY (contain WRKY15, WRKY2, WRKY57, WRKY13, WRKY6, and PER40) were strongly induced by suboptimal temperature stress. AP2/ERF was strongly induced by both suboptimal and low-temperature stresses in cucumber roots. Furthermore, we found that CBF1 responded mainly to suboptimal temperature stress. WRKY6 is induced by freezing stress in apple branches (Xu et al., 2023). In cucumbers, CsWRKY21 and CsWRKY46 positively regulate the response to cold stress in different ways. CsWRKY21 is involved in the CBF-mediated cold response signaling pathway. CsWRKY46 enhances the expression of ABI5, a key transcription factor in the ABA signaling pathway. This activates the expression of RD29A and COR47 and participates in the ABA-dependent cold response regulation pathway (Ying et al., 2016). Therefore, it is important to analyze the expression patterns of AP2/ERF and WRKY in cucumber roots under low-temperature stress as well as the molecular expression networks of key genes regulated by these TFs.

Cold response signaling pathways in plants are distinguished by whether the regulation of signal transduction relies on CBF. Numerous studies have demonstrated that CBFs proteins play a central role in cold acclimation. Cold-responsive signals in plants are mainly regulated by the ICE-CBF-COR (ICE, CBF expression protein inducer; COR, cold-responsive gene) mode (Ramezani et al., 2017; Tang et al., 2022). The transcription level was significantly upregulated by ICE. It also activates the expression of downstream cold-responsive genes (CORs) by binding to the cis-elements in their promoters (Liu et al., 2018). However, current studies show that some TFs in the AP2/ERF and WRKY families are involved in the regulation of plant hypothermia responses through a pathway that is independent of CBF. In this study, the CBF transcription factor (*CsaV33G016760*), which had been identified in cucumber, was downregulated in cucumber roots in response to low-temperature stress. However, most DETFs were upregulated in the AP2 family and were strongly induced by both suboptimal and low-temperature stresses. This suggests that the regulatory pathway of the response to low-temperature stress in cucumber roots mainly depends on the AP2/ERF family, without dependence on CBF. However, this conclusion requires further investigation.

## 5 Conclusion

In the present study, cucumber cultivars were subjected to cold stress at different temperatures. By combining transcriptomics and metabolomics data, DEGs and DEMs responding to chilling stress were identified in the cucumber root samples RR, SR, and LR. Hormone detection indicated that auxin had the highest content in the roots. Thus, JA may play an important role in low-temperature stress. Furthermore, under chilling stress, the highest phenolic acid content was observed in the cucumber roots. The main phenolic acids accumulated in cucumber roots are p-coumaryl alcohol, benzaldehyde, and isoferulic acid. The downward trends of plumerane and alkaloids with temperature were similar. The organic acid content increased sharply with decreasing temperature. 2-Isopropylmalic acid, iminodiacetic acid, and JAs are the main organic acids accumulated in cucumber roots. Additionally, triterpenes may play an important role in chilling resistance. DEGs and DEMs were significantly enriched in benzoxazinoid biosynthesis in the RR vs. SR and RR vs. LR groups. DEGs and DEMs were significantly enriched in phenylpropanoid biosynthesis in the RR vs. LR and SR vs. LR groups. Most DETFs in the AP2/ERF family were strongly induced by both suboptimal and low-temperature stresses in cucumber roots. In conclusion, these results explain the response to chilling stress in cucumber roots, but further studies on the differential expression patterns of the components of these pathways are needed to reveal the mechanisms of low temperatures in cucumbers.

## Data Availability

Original datasets are available in a publicly accessible repository: The original contributions presented in the study are publicly available. This data can be found at the NCBI with accession number: PRJNA1135659.
