# Supplementary material for: Comparison of transcriptome and metabolome analysis revealed cold-resistant metabolic pathways in cucumber roots under low-temperature stress in root zone
Source: Front Plant Sci. 2024 Sep 9;15:1413716. doi: 10.3389/fpls.2024.1413716 (PMC11416975; doi:10.3389/fpls.2024.1413716)
Supplement: Supplementary file 1 [file DataSheet1.docx]

**
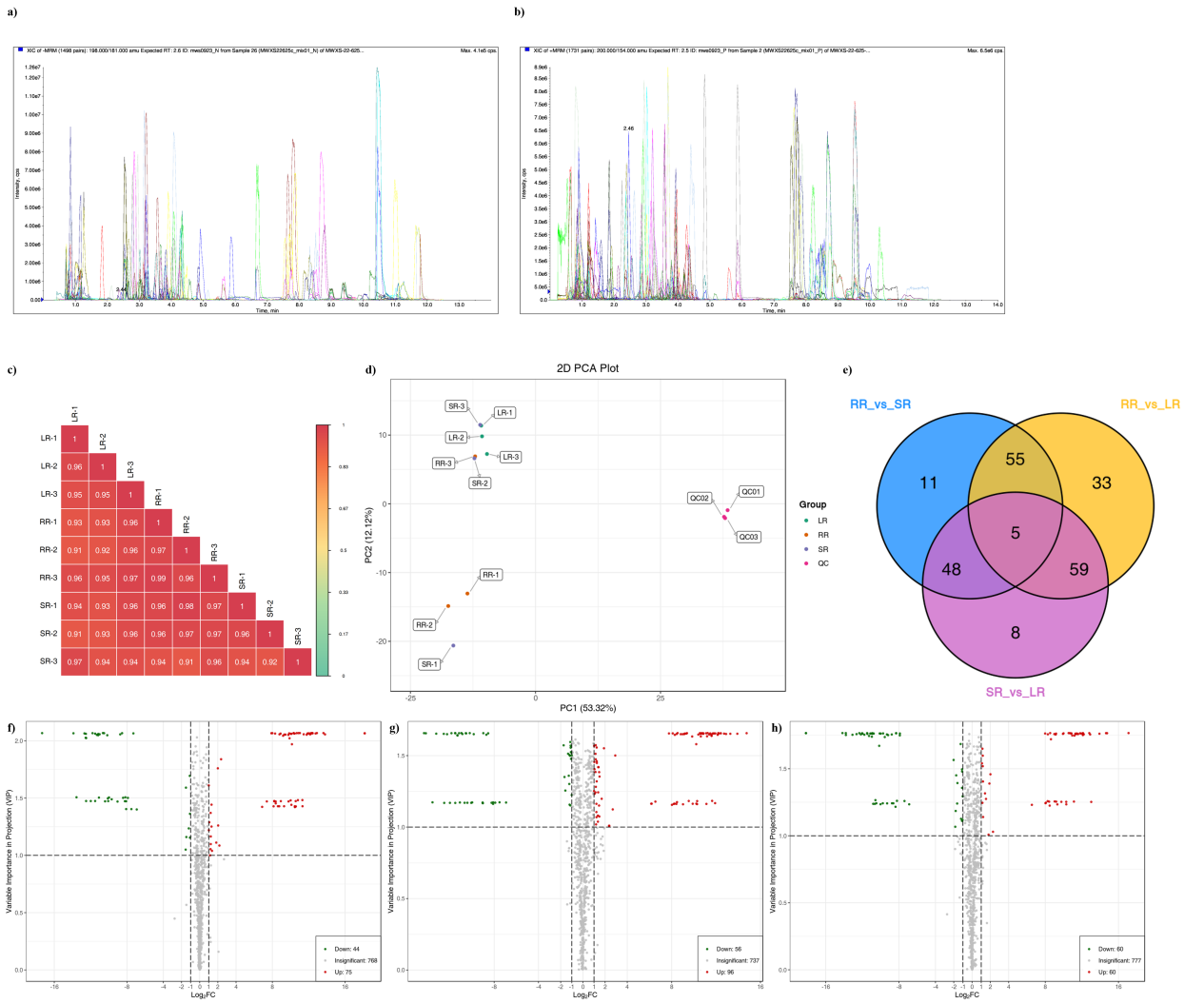
**

**Supplementary Figure 1 MRM detection of multimodal maps-N (a) and P (b) of cucumber root sample. Correlation (c) and principal component analysis (d) between repeat root samples of cucumber. Venn diagram (e) depicting the shared and specific metabolites in the RR, SR and LR. DAM Volcano Map of RR_vs_SR (f), RR_vs_LR (g) and SR_vs_LR (h). RR: The root of cucumber in room temperature condition. SR: The root of cucumber in suboptimal temperature condition LR: The root of cucumber in low temperature condition.**
