# Supplementary material for: Comparison of transcriptome and metabolome analysis revealed cold-resistant metabolic pathways in cucumber roots under low-temperature stress in root zone
Source: Front Plant Sci. 2024 Sep 9;15:1413716. doi: 10.3389/fpls.2024.1413716 (PMC11416975; doi:10.3389/fpls.2024.1413716)
Supplement: Supplementary file 2 [file DataSheet2.docx]

**
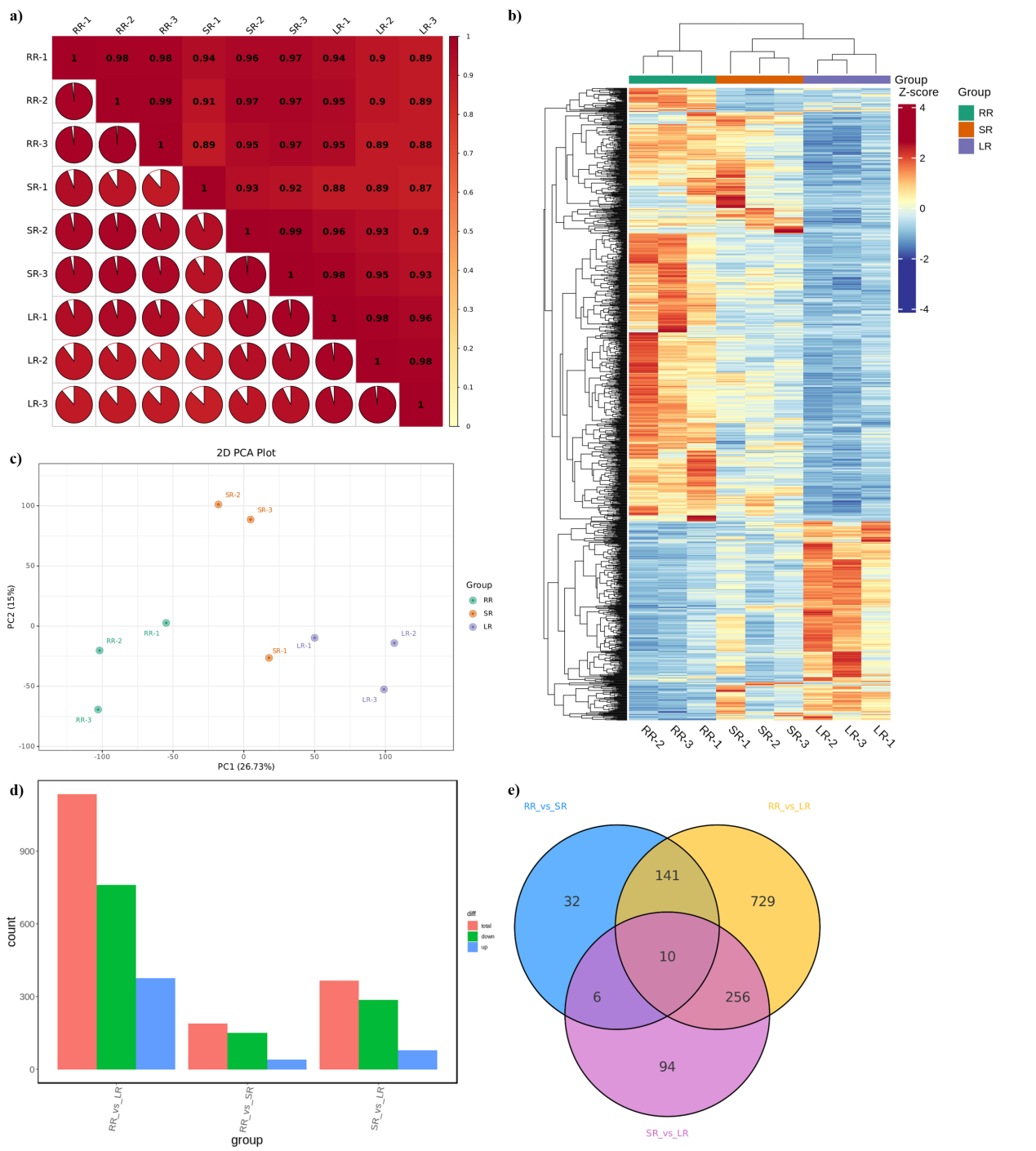
**

**Supplementary Figure 2 Correlation (a), cluster heatmap (b) and principal component analysis (c) between RNA-seq repeat root samples of cucumber. The statistics of differential genes in root of cucumber d): The statistics of differential genes in root, the vertical axis represents the number of differential genes; Bar values are average on three replicates. Venn diagram (e) depicting the shared and specific genes in the RR, SR and LR. RR: The root of cucumber in room temperature condition. SR: The root of cucumber in suboptimal temperature condition LR: The root of cucumber in low temperature condition.**
